# Functional evaluation of sublingual microcirculation indicates successful weaning from VA-ECMO in cardiogenic shock

**DOI:** 10.1186/s13054-017-1855-2

**Published:** 2017-10-26

**Authors:** Sakir Akin, Dinis dos Reis Miranda, Kadir Caliskan, Osama I. Soliman, Goksel Guven, Ard Struijs, Robert J. van Thiel, Lucia S. Jewbali, Alexandre Lima, Diederik Gommers, Felix Zijlstra, Can Ince

**Affiliations:** 1 Department of Intensive Care, Erasmus MC, University Medical Center Rotterdam, Room H-603a, ‘s-Gravendijkwal 230, 3015 CE Rotterdam, The Netherlands; 2 Department of Cardiology, Erasmus MC, University Medical Center Rotterdam, Room H-603a, ‘s-Gravendijkwal 230, 3015 CE Rotterdam, The Netherlands

**Keywords:** Cardiogenic shock, VA-ECMO, Microcirculation, Incident dark field imaging, Sublingual, CytoCam, Weaning, Cardiac recovery

## Abstract

**Background:**

Veno-arterial extracorporeal membrane oxygenation (VA-ECMO) is increasingly adopted for the treatment of cardiogenic shock (CS). However, a marker of successful weaning remains largely unknown. Our hypothesis was that successful weaning is associated with sustained microcirculatory function during ECMO flow reduction. Therefore, we sought to test the usefulness of microcirculatory imaging in the same sublingual spot, using incident dark field (IDF) imaging in assessing successful weaning from VA-ECMO and compare IDF imaging with echocardiographic parameters.

**Methods:**

Weaning was performed by decreasing the VA-ECMO flow to 50% (F_50_) from the baseline. The endpoint of the study was successful VA-ECMO explantation within 48 hours after weaning. The response of sublingual microcirculation to a weaning attempt (WA) was evaluated. Microcirculation was measured in one sublingual area (single spot (ss)) using CytoCam IDF imaging during WA. Total vessel density (TVDss) and perfused vessel density (PVDss) of the sublingual area were evaluated before and during 50% flow reduction (TVDss_F50_, PVDss_F50_) after a WA and compared to conventional echocardiographic parameters as indicators of the success or failure of the WA.

**Results:**

Patients (n = 13) aged 49 ± 18 years, who received VA-ECMO for the treatment of refractory CS due to pulmonary embolism (n = 5), post cardiotomy (n = 3), acute coronary syndrome (n = 2), myocarditis (n = 2) and drug intoxication (n = 1), were included. TVDss_F50_ (21.9 vs 12.9 mm/mm^2^, *p* = 0.001), PVDss_F50_ (19.7 vs 12.4 mm/mm^2^, *p* = 0.01) and aortic velocity–time integral (VTI) at 50% flow reduction (VTI_F50_) were higher in patients successfully weaned vs not successfully weaned. The area under the curve (AUC) was 0.99 vs 0.93 vs 0.85 for TVDss_F50_ (small vessels) >12.2 mm/mm^2^, left ventricular ejection fraction (LVEF) >15% and aortic VTI >11 cm. Likewise, the AUC was 0.91 vs 0.93 vs 0.85 for the PVDss_F50_ (all vessels) >14.8 mm/mm^2^, LVEF >15% and aortic VTI >11 cm.

**Conclusion:**

This study identified sublingual microcirculation as a novel potential marker for identifying successful weaning from VA-ECMO. Sustained values of TVDss_F50_ and PVDss_F50_ were found to be specific and sensitive indicators of successful weaning from VA-ECMO as compared to echocardiographic parameters.

**Electronic supplementary material:**

The online version of this article (doi:10.1186/s13054-017-1855-2) contains supplementary material, which is available to authorized users.

## Background

Cardiogenic shock (CS) associated with cardiac pump failure results in a state of inadequate tissue perfusion, which leads to organ failure with a mortality rate between 50 and 80% [1]. Veno-arterial extracorporeal membrane oxygenation (VA-ECMO) is considered a lifesaving treatment that is increasingly used for the treatment of critically ill patients that have experienced CS [2–4]. However, mortality rates remain high, with reports of up to 44% mortality despite the use of VA-ECMO [1, 5].

Current strategies for weaning from VA-ECMO are ongoing, largely unknown and based on empirical evidence [6–8]. Most of the current markers of weaning from VA-ECMO are based on echocardiography, such as aortic velocity–time integral (VTI), left ventricular ejection fraction (LVEF), and tissue Doppler lateral mitral annulus peak systolic velocity (TDSa) [9]. However, performing high-quality echocardiography in critically ill patients requires specialized training and is relatively costly [10].

In a recent study, we found that the initial inability of VA-ECMO to recruit the microcirculatory alteration associated with CS predicts adverse outcomes following VA-ECMO treatment irrespective of improved systemic hemodynamic parameters [11]. This is based on the concept that the success of resuscitation from states of circulatory shock is the normalization of microcirculatory and tissue perfusion [12, 13].

It is known that there is possibly a loss of coherence between the systemic and microcirculation, which can occur in states of shock and resuscitation [14]. Previous studies measuring sublingual microcirculation using hand-held video microscopy have shown impairment of sublingual microcirculation to be associated with CS [12, 13, 15]. In addition, studies have found that sustained microcirculatory perfusion by VA-ECMO as detected by handheld video microscopes is associated with lower morbidity and even mortality [12, 14].

Our hypothesis was that successful weaning is associated with sustained microcirculatory function during ECMO flow reduction. Therefore, we sought to test the usefulness of microcirculatory imaging in the same sublingual spot, using incident dark field (IDF) imaging in assessing successful weaning from VA-ECMO and to compare IDF imaging with echocardiographic parameters [7].

## Methods

### Study population

**Fig. 1:**
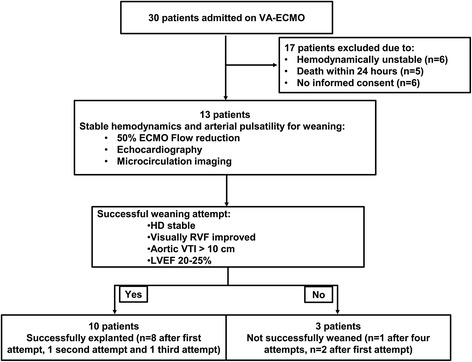
Flow chart for inclusion for weaning attempts and their outcomes (n = 13). VA-ECMO, veno-arterial extracorporeal membrane oxygenation; VTI, velocity − time integral; LVEF, left ventricular ejection fraction; HD, Hemodynamic; RVF, Right ventricular function

The institutional medical ethics board of the Erasmus Medical Center approved this study under protocol number NL45915.078.13, and informed consent was obtained from all patients and/or legal representatives. Between October 2014 and January 2016, our prospective observational study included all eligible patients under VA-ECMO support admitted to the Intensive Care Unit of our center, a tertiary and national referral center for end-stage heart failure, heart transplantation and left ventricular assist devices. Inclusion criteria were > 18 years old and need for VA-ECMO due to any form of refractory cardiogenic shock. Exclusion criteria were being moribund and refusal to participate in the study (Fig. 1).

### Echocardiography

#### Acquisition

Transthoracic echocardiography (TTE) and/or transesophageal (TEE) echocardiography was performed during weaning attempts using the CX50 ultrasound system (Philips Medical System, Best, The Netherlands). Pulsed-wave and continuous-wave Doppler signals were recorded at a sweep speed of 50–100 mm/s. Color Doppler recordings were optimized for display with the color velocity scale at ± 60 (50–70 cm/s) during the entire study.

#### Analysis

All echocardiograms were analyzed by two experienced echo cardiologists (OS and SA), in accordance with published guidelines [16], using the QLAB quantification software (Philips Healthcare, Best, The Netherlands). Aortic VTI was measured by manually tracing the spectral envelope of continuous-wave Doppler in the apical 5-chamber or 3-chamber view. The LVEF was visually estimated from apical views. The right ventricular function was assessed by measuring the tricuspid annular planer systolic excursion (TAPSE) from the M-mode images in the apical 4-chamber view. Tissue Doppler lateral mitral annulus peak systolic velocity (TDSa) was also measured when feasible.

#### Weaning strategy

Weaning from VA-ECMO was initiated in patients with persistently stable hemodynamics (mean arterial pressure > 60 mmHg, lactate < 2 mmol/L and mixed venous saturation values > 65%) and with persisting arterial pulsatility wave on the monitor under low doses of inotropic support (Fig. 1). Weaning was performed by lowering the blood flow to 50% of the baseline value under hemodynamic and echocardiographic surveillance. Persisting hemodynamic stability was defined as aortic VTI > 10 cm and estimated LVEF > 20–25% [7, 9]. Patients who recovered from severe cardiac dysfunction and who tolerated the weaning attempt were considered for device removal [7].

#### Microcirculatory imaging

##### Acquisition

Sublingual microcirculation was measured independently of echocardiography data during the same weaning attempt, just before or after echocardiography, at baseline (100%) VA-ECMO flow (F100), and after reducing VA-ECMO flow to 50% of the baseline flow (F50) and at returning VA-ECMO flow to baseline after 2 minutes (F100). We performed these measurements just before or after the classical weaning attempt using echocardiography. These microcirculatory data were not used to drive ECMO management.

Such a weaning attempt was followed by microcirculatory measurements during a weaning attempt, which took a maximum of 10 minutes in total. All aspects of microcirculatory measurements were performed as stable video recordings with a duration of 3–5 seconds by placing the CytoCam IDF imaging camera (Braedius Medical, Huizen, The Netherlands) [17] in the same sublingual area during the entire procedure, with total vessel density measured at a single spot (TVD_SS_).

The IDF device consists of a computer-controlled, high-resolution image sensor in combination with a specifically designed microscope lens at the end of an image guide, covered by a disposable sterile cap. Placing the tip of the guide to the sublingual tissue surface provides high-resolution images of the microcirculation where red blood cells can be clearly visualized flowing through the microvessels.

**Fig. 2:**
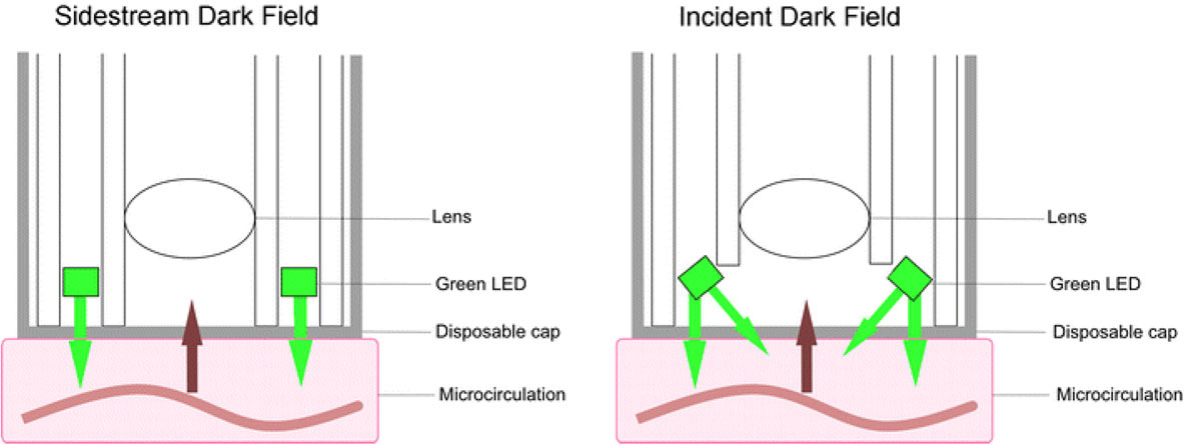
Technical differences between sidestream dark field (SDF) and incident dark field (IDF) technique adapted with permission from reference [19]. LED, light-emitting diode

This device is based on IDF imaging technique as described by Sherman et al. [18]. Sidestream dark field (SDF) technique optically isolates the incoming light from the reflected while IDF illuminates the field in a non-homogeneous fashion according to dark field. In this CytoCam device, there are prominent technical improvements such as digital signal, lower weight, and higher optical resolutions, compared to previous devices. In Fig. 2 and Additional file 1: Table S1 we show the visual and characteristic differences between SDF and IDF technique as adapted. from van Elteren et al. [19].

This new iteration of the device with improved optics detects 30% more sublingual vessels than the previous generation microscope [17, 19, 20]. Microcirculatory parameters are quantified by analyzing the movies using specialized image processing software (Automated Vascular Analysis (AVA)) [21].

#### Analysis

Two microcirculation experts (SA and GG) independently analyzed all microcirculation parameters based on international consensus on the quantification of sublingual microcirculatory alterations [22]. The images were analyzed to determine the functional parameters of large microvessels (> 25 μm) and small vessels (≤ 25 μm). These parameters consisted of the TVD (mm/mm^2^); perfused vessel density (PVD (mm/mm^2^)); and proportion of perfused vessels (PPV (%)) in accordance with international consensus guidelines related to the quantification of such microcirculatory images [22]. Microcirculatory measurements were compared with echocardiography parameters (LVEF, aortic VTI and TDSa, if available) and used to evaluate the last weaning procedure as described by Aissaoui et al. [7].

#### Statistics

Categorical variables are presented as frequencies and percentages. Continuous variables are presented as the mean ± standard deviation (SD). Continuous variables were compared with the Mann-Whitney U test. For comparisons within the same group, the microcirculatory parameters of patients at different time points were analyzed using the Friedman and Wilcoxon test. To compare the microcirculatory parameters of patients successfully weaned (SW) and not successfully weaned (NSW) at different time points, the generalized linear model repeated measurements test was used. Spearman’s correlation analysis was used to compare the correlation between echocardiographic and microcirculatory parameters in SW and NSW patients. Statistical significance was defined by a *p* value <0.05. Analyses were performed using SPSS version 21.0.0.1 (SPSS, IBM, Armonk, NY, USA) and MedCalc (MedCalc, Ostend, Belgium) software.

## Results

The study population consisted of 13 patients with cardiogenic shock, 49 ± 18 years old, 11 of whom (85%) were male. Of the 13 patients, 10 were SW from VA-ECMO (Fig. 1). The causes of admission were pulmonary embolism (n = 5, 38%), post cardiotomy (n = 3, 23%), acute coronary syndrome (n = 2, 15%), myocarditis (n = 2, 15%) and drug intoxication (n = 1, 8%) (Table 1). In total, 19 weaning attempts were performed in 13 patients, of which 10 were successful and led to cardiac recovery (CR): 8 out of 10 were SW after the first attempt. Five patients need more than one weaning attempt (Fig. 1). In the nine unsuccessful weaning attempts, three patients died (non-cardiac recovery; NCR) after six unsuccessful weaning attempts and two patients were SW after two and three attempts, respectively.

**Table 1:** Baseline characteristics of the microcirculation of 13 patients observed during weaning attempts

Baseline characteristics	Successful weaning (n = 10)	Non-successful weaning (n = 3)	Total	*p* value
Number of patients in each group	10	3	13	*0.02*
Weaning attempts	13	6	19	0.32
Age	56 ± 17	41 ± 16	49 ± 18	0.43
Male gender	9	2	11	0.11
Etiology				
PE	5	0	5	*0.03*
Post cardiotomy	2	1	3	1.0
ACS	1	1	2	1.0
Myocarditis	1	1	2	1.0
Intoxication	1	0	1	1.0
Days on ICU	15 (6–65)	30 (3–36)	20 (3–65)	0.72
Days of ECMO	5 (2–21)	21 (3–36)	13 (2–36)	*0.02*
WA p.p.	1.3 ± 0.7	2.0 ± 1.7	1.5 ± 0.5	0.21

*Abbreviations*: *ACS* acute coronary syndrome, *ICU* intensive care unit, *PE* pulmonary embolism, *VA-ECMO* veno-arterial extracorporeal membrane oxygenation, *p.p.* per patient, *WA* weaning attempts

*p* values in italics are statistically significant

The global hemodynamics were not significantly different between SW and NSW patients during weaning attempts (Additional file 2: Figure S1A and S1B). Successful and unsuccessful weaning was classified according to echocardiographic assessment as described previously. The results from the microcirculation measurements showed that in SW patients, TVDss, PVDss and PPVss measured in the same sublingual area maintained their values prior to the weaning attempt, whereas these values decreased in the patients who were NSW (Additional file 3: Figure S2A through Additional file 4: Figure S2F). TVDss, PVDss and PPVss were statistically significantly reduced following a flow reduction (from F_100_ to F_50_) in patients who were not NSW (TVDss all vessels *p* = 0.001; PVDss all vessels *p* = 0.01; PPVss all vessels *p* = 0.04; TVD small vessels *p* = 0.001; PVDss small vessels *p* = 0.01; PPV small vessels *p* = 0.03).

**Fig. 3:**
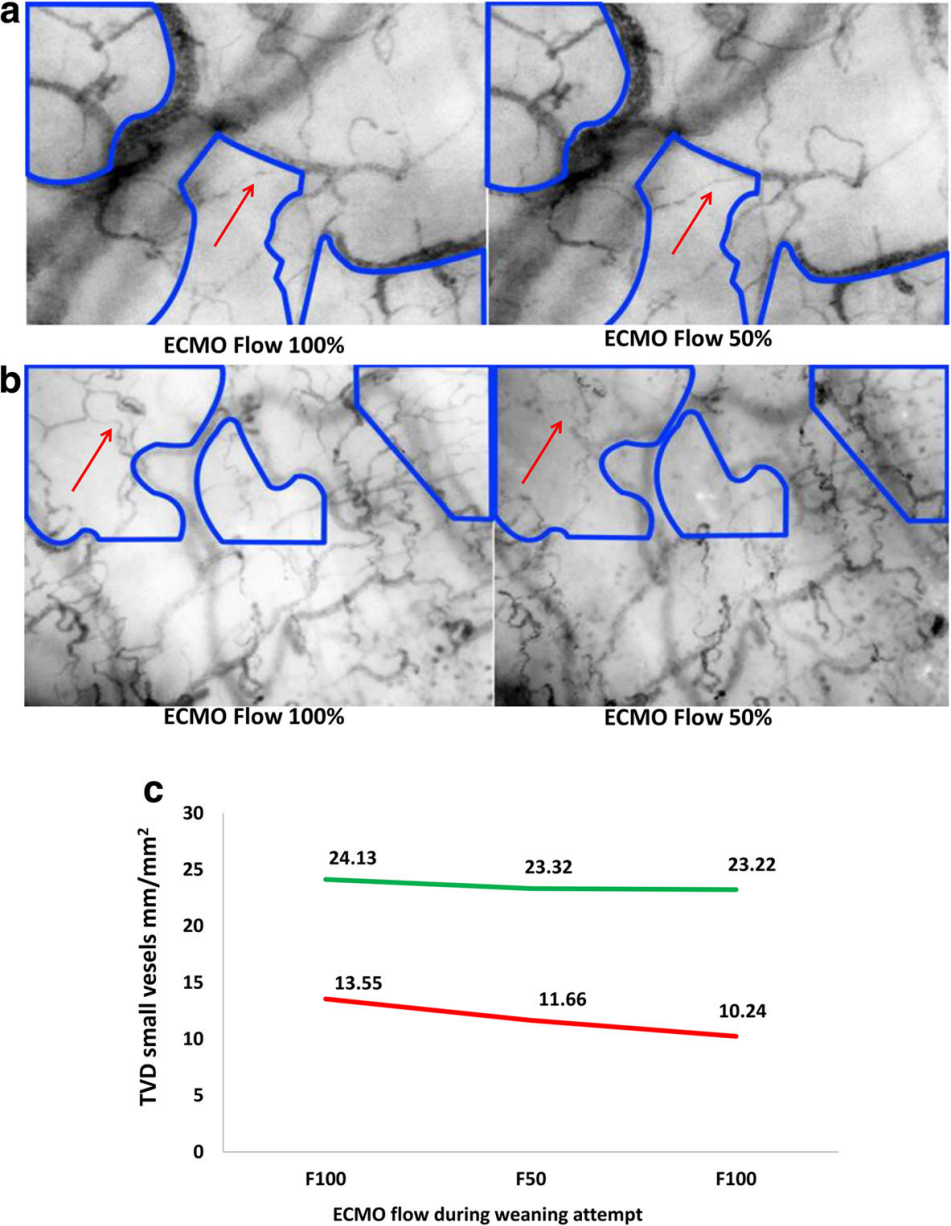
**a** Microcirculatory alterations in successfully weaned (SW) vs not successfully weaned (NSW) patients during weaning attempts with baseline flow (F_100_) and 50% of the baseline flow (F_50_). Examples are shown of microcirculation in the same sublingual area in two patients during F_100_ and F_50_. Images were taken from a 51-year-old man, there was cardiac recovery 3 days after tentamen suicidii with 900 mg of amlodipine and 1600 mg of hydrochlorothiazide in the same sublingual area during a weaning attempt on day 3, with no alterations in microcirculation (F_100_, veno-arterial extracorporeal membrane oxygenation (VA-ECMO) flow 6.1 L/min; mean arterial pressure (MAP) 77 mmHg and F_50_, VA-ECMO flow 3 L/min; MAP 74 mmHg). **b** Images were taken from a 26-year-old woman with stable human immunodeficiency virus infection (HIV) developed myocarditis and biventricular heart failure. After 4 weeks, there was no improvement in cardiac function. The microcirculatory images were documented during four weaning attempts without improvement. The same sublingual area was also used during the last weaning attempt, which showed obvious persisting deterioration in microcirculation (blue zones) (F_100_, VA-ECMO flow 4.7 L/min; MAP 75 mmHg and F_50_, VA-ECMO flow 2.7 L/min; MAP 67 mmHg). **c** Changes in total vessel density (TVD) in small vessels during non-successful (day 2, red) and successful (day 4, green) weaning attempts in patient 7. This 65-year-old man suffered from cardiogenic shock after coronary artery bypass graft (CABG) surgery

The images acquired from the same sublingual area during VA-ECMO 100% and 50% flow, were evaluated visually and then quantified side by side by comparing total small and large vessel densities (Fig. 3a and b see also Additional file 5: Clip 1 and Additional file 6: Clip 2). In SW patients, no or minimal alterations in microcirculation were seen during VA-ECMO 100% and 50% flow. In contrast, patients who were not SW had clear deterioration in total small and large vessel densities during VA-ECMO 50% flow (Additional file 7: Table S2A, B and C). Figure 3c shows an example of successful and not successful weaning attempts in the same patient.

TVD of all vessels and TVD of small vessels were statistically reduced in the NSW patients during 50% VA-ECMO flow compared to no change or even increased values in SW patients. Examples of the recorded moving images of the sublingual microcirculation of the two categories of patients can be found in Additional file 8.

A comparison of the microcirculatory parameters with echocardiographic parameters values according to the published Aissaoui criteria for weaning from VA-ECMO [9] showed good correlation, especially with LVEF (*r* = 0.6214 and *p* = 0.01) (Additional file 7: Table S3).

Receiver operating characteristic (ROC) curves showed the area under the curve (AUC) was 0.99 vs 0.93 vs 0.85 for the TVDss_F50_ (small vessels) >12.2 mm/mm^2^, LVEF > 15% and aortic VTI > 11 cm (Additional file 9: Figure S3). Likewise, the AUC was 0.91 vs 0.93 vs 0.85 for the PVDss_F50_ (all vessels) > 14.8 mm/mm^2^, LVEF > 15% and aortic VTI > 11 cm.

**Fig. 4:**
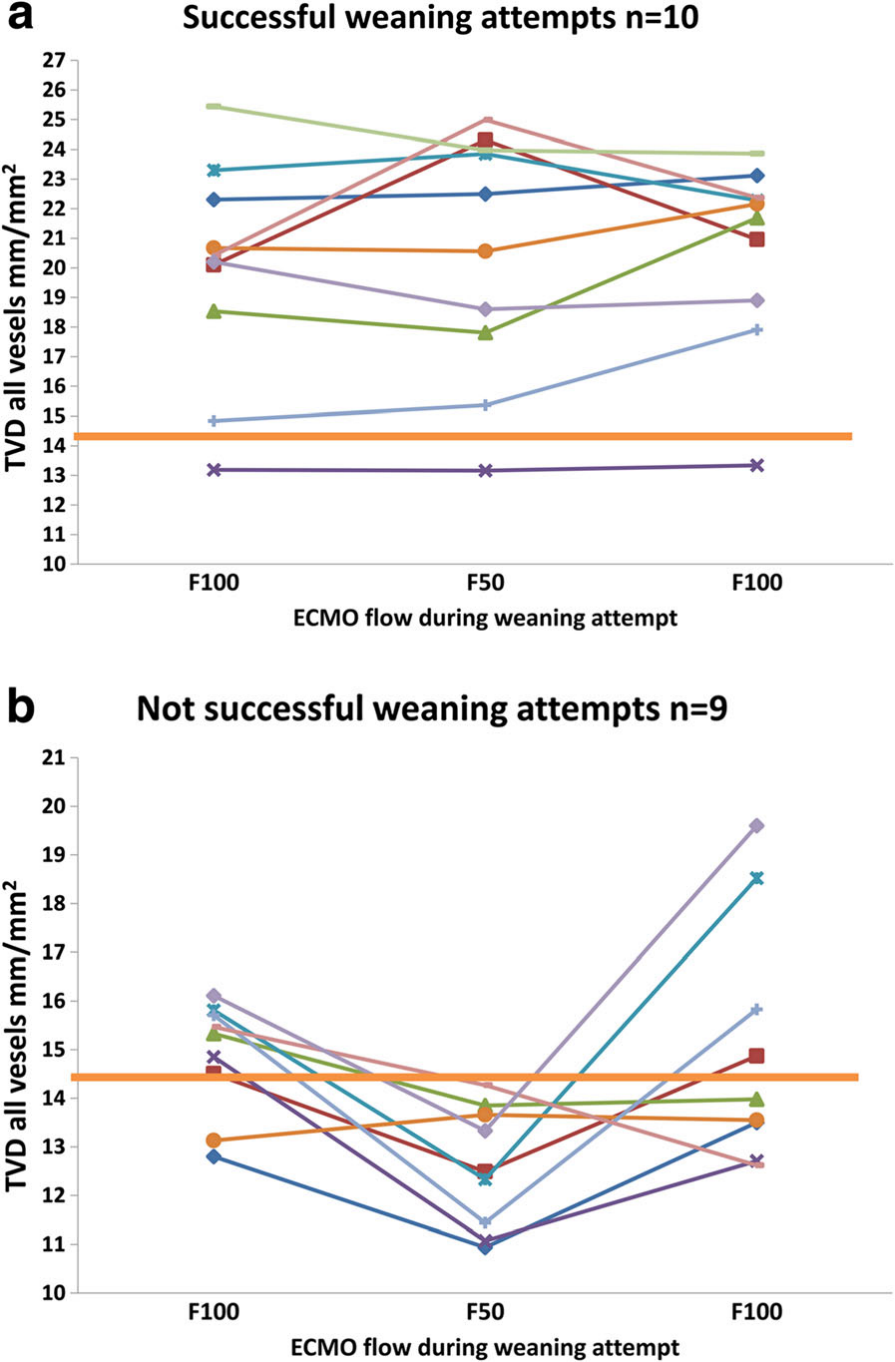
**a** Data from individual successful weaning attempts (n = 10), in which 8 demonstrated sustained or increased total vessel density (TVD) independently from extracorporeal membrane oxygenation (ECMO) flow. Two weaning attempts showed decreased TVD after reducing the ECMO flow. **b** Data from individual unsuccessful weaning attempts (n = 9), in which 8 demonstrated non-sustained or declining TVD during 50% ECMO flow. One weaning attempt showed sustained TVD after reducing the ECMO flow in contrast to clinical judgment. However, the TVD was lower than the cutoff 14.3 mm/mm2

After reanalyzing the data using the individual outcome of weaning attempts as an endpoint, TVD was highly predictive of a successful weaning attempt from ECMO (Additional file 7: Table S4A and S4B). In three weaning attempts, the outcome of SW and NSW patients did not match with the TVD changes in the microcirculation, with false negative (n = 2) and false positive (n = 1) values seen. The positive predictive value of sustained microcirculation for successful weaning is 89%. Two of these three attempts were in the same patient. All unsuccessful weaning attempts were during 50% ECMO flow below the 14.3 mm/mm^2^ cutoff point of TVDss_F50_ predicting successful weaning, when pooled to determine the success of each weaning attempt separately (Fig. 4a and b).

## Discussion

The main finding in this study is that sustained sublingual microcirculation during VA-ECMO flow reduction in a convenient cohort sample of patients supported with VA-ECMO during cardiogenic shock, can provide a marker for the success of weaning from VA-ECMO. Cardiogenic shock affects all organs and compromises central hemodynamic cardiovascular function and consequently tissue perfusion. Currently used strategies for weaning from VA-ECMO are largely based on echocardiographic parameters. However, performing echocardiography in the ICU is challenging [10]. The results of the present study show that functional parameters of microcirculation, including TVDss and PVDss, reflect recovery from cardiogenic shock and predict successful weaning from VA-ECMO. Patients who were successfully weaned had significantly higher baseline TVDss and PVDss compared to those of patients who were not successfully weaned. Even though global hemodynamics were comparable between patients with and without successful weaning, microcirculatory parameters were significantly different. The occurrence of such disassociation between macro-circulation and microcirculation, referred to as a loss of hemodynamic coherence, has been described before in other conditions of cardiovascular compromise [11, 23–28].

The microcirculatory approach presented in this study could provide an alternative approach for the rapid assessment of cardiac recovery from cardiogenic shock during weaning attempts and/or in addition to echocardiographic evaluation. This approach could also be useful for the echocardiographic assessment of the left ventricular systolic function, especially in ICU patients with poor echo windows. Cavarocchi et al. [8] used a 4-stage strategy to evaluate 50% VA-ECMO blood flow, volume challenge and inotropic challenge during at least 1 hour of the continual monitoring of heart rate, blood pressure, and right ventricle (RV) and left ventricle (LV) function under transesophageal echocardiography. Weaning was considered successful when both LV and RV functions tolerated volume challenge and demonstrated inotropic reserve. However, this strategy required intravenous sedation to tolerate transesophageal echocardiography and an increased physical load in non-intubated patients throughout the weaning attempt with need for continuous therapeutic anticoagulation. A different approach involves the use of biomarkers as indicators of the success of weaning; however, such markers appear very late and seem inconclusive for determining the success of weaning from VA-ECMO. The usefulness of biomarkers in weaning from VA-ECMO, therefore, remains controversial. In line with this limitation, Luyt et al. [29] reported that in patients with refractory cardiogenic shock receiving VA-ECMO support, early measurements of cardiac biomarkers are not useful for identifying those who would recover.

In a VA ECMO weaning study, Aissaoui et al. measured left ventricular functional parameters (e.g., LVEF, VTI, TDSa) and found these to be good predictors of successful weaning. However, such echocardiographic assessment is limited to the evaluation of the left heart function under the condition that there are sufficient windows for analysis [9]. The echocardiography parameters used in these weaning attempts, however, do not provide information about the right heart function, systemic hemodynamics or tissue perfusion, which also deteriorate as a consequence of cardiogenic shock. Our study shows that during weaning attempts, recovery from cardiogenic shock is revealed in the microcirculation, which agrees with total cardiac recovery upon echocardiography.

Monitoring the microcirculation using direct vital imaging with handheld microscopy has the potential to be the technique of choice to assess tissue perfusion in different phases of shock [27, 30]. The study described in this paper illustrated that the assessment of sublingual microcirculation and the echocardiographic evaluation of cardiac function was acceptably matched in discriminating between patients who were and were not successfully weaned. Microcirculatory evaluation was rapid since alterations were observed within 2 minutes following a lowering of ECMO flow.

Physiologically, this fast-adapting mechanism can also be evaluated from fractional flow reserve (FFR) measurements in coronary angiography. In these measurements, the hyperemic phase after the resolution of stenosis also occurs within 2 minutes [31]. Several studies have been performed on microcirculatory alterations during ECMO [32, 33] with differing results concerning the relationship between global hemodynamics to the microcirculation [23, 34–38]. In a recent study, however, we found a significant predictive value of sublingual measured perfused vessel density in VA-ECMO patients for survival in the ICU [11]. However, to date, sublingual measurements have not been employed to guide weaning from VA-ECMO.

### Limitations

The authors acknowledge the following limitations. First, this was a single-center observational study with a small population of patients with various underlying diseases causing cardiogenic shock. Furthermore, analyses of echocardiography and global hemodynamics, together with microcirculatory parameters measured in the same sublingual area, were performed only in patients deemed eligible to wean. This meant that we could not perform microcirculatory measurements without echocardiography for weaning attempts in patients under VA-ECMO support because of the observational nature.

The methods of measurement and evaluation of the microcirculation remain sensitive to artifacts and technical limitations. The strength of our study from a methodological perspective, however, lies in the fact that all measurements were performed in the same area (single spot). This methodology allowed us to assess the response of single microvessels to changes in pump settings as against comparing the mean value of microcirculation parameters of images at different locations and at different time points.

## Conclusion

We found that the functional microcirculatory parameters measured sublingually using IDF imaging (TVDss_F50_ and PVDss_F50_) during weaning attempts for patients from VA-ECMO showed essential alterations within 2 minutes and prediction of cardiac recovery after cardiogenic shock. Future clinical and possible crossover studies should be designed in larger study populations undergoing VA-ECMO for monitoring microcirculation to guide weaning attempts.

## Key messages

- Veno-arterial extracorporeal membrane oxygenation (VA-ECMO) use is a last option for survival in many types of cardiogenic shock (CS).
- Conventional weaning from VA-ECMO is guided by echocardiographic parameters such as the aortic velocity − time integral on continuous wave Doppler recordings from left ventricular outflow tract and by assessing improvement in left ventricular ejection fraction. Echocardiographic measurements are not easily obtained in the ICU settings. On the other hand, a novel imaging technique, dark field imaging of the microcirculation, is quite feasible in almost all patients in the ICU.
- Identified sublingual microcirculation is a novel potential marker for identifying successful weaning from VA-ECMO. Sustained values of single-spot measurements of total vessel density during 50% flow reduction (TVDss_F50_) and single-spot measurements of perfused vessel density during 50% flow reduction (PVDss_F50_) were found to be specific and sensitive indicators of successful weaning from VA-ECMO as compared to echocardiographic parameters. Therefore, weaning from VA-ECMO could be performed by imaging of the microcirculation using simple markers of tissue perfusion during weaning attempts.

## Additional files

Additional file 1: Table S1. Shows technical overview of the SDF and IDF devices (adapted with permission from van Elteren et al. [19]). (DOCX 12 kb)

Additional file 2: Figure S1. **a** Mean arterial pressure (MAP) and **b** heart rate (HR) in patients successfully weaned (SW) and not successfully weaned (NSW). (TIF 2047 kb)

Additional file 3: Figure S2. **a** Total vessel density (TVD), **b** perfused vessel density (PVD) and **c** portion of the perfused vessels (PPV) in all vessels (length between 25 and 100 μm) at flow time points of 100% ECMO flow (F100) and 50% ECMO flow (F50) are compared between patients successfully and not successfully weaned (SW and NSW, respectively). (TIF 3043 kb)

Additional file 4: Figure S2. **a** TVD, **b** PVD and **c** PPV in small vessels (capillaries < 25 μm) were compared between each patient during weaning attempts at flow time points of 100% ECMO flow (F100) and 50% ECMO flow (F50) are compared between patients successfully and not successfully weaned (SW and NSW, respectively). (TIF 2730 kb)

Additional file 5: Clip 1 shows movie of a patient with successful weaning attempt. Microcirculatory image clips recorded during a weaning attempt. (MOV 3029 kb)

Additional file 6: Clip 2 shows movie of a patient with non-successful weaning attempt. Microcirculatory image clips recorded during a weaning attempt. (MP4 749 kb)

Additional file 7: Table S2A. Shows microcirculatory changes during weaning attempts. **Table S2B.** Shows microcirculatory differences at baseline VA-ECMO flow. **Table S2C.** shows microcirculatory differences between baseline and reduced VA-ECMO flow. **Table S3.** Shows correlation between microcirculatory and echocardiographic parameters for the prediction of successful weaning from VA-ECMO. **Table S4A.** Shows comparison of the ROC curves of microcirculatory and echocardiographic parameters for the prediction of successful weaning from VA-ECMO. **Table S4B.** Shows comparison of the ROC curves of microcirculatory and echocardiographic parameters for the prediction of successful weaning from VA-ECMO. (DOCX 27 kb)

Additional file 8: Additionale data. (DOCX 13 kb)

Additional file 9: Figure S3. Receiver operating characteristic (ROC) curves for significantly different values from microcirculation and echocardiography as the best parameter from microcirculation and echocardiography according to the area under the ROC curve (AUC). A cutoff value of TVDss_F50_ > 12.2 mm/mm^2^ (small vessels) has a higher sensitivity, specificity, and AUC (0.99, 95% CI (0.78–1.00) vs 0.85, 95% CI (0.596–0.97)) compared with aortic VTI _F50_ > 11 cm and compared with LVEF >15% (0.99, 95% CI (0.78–1.00) vs 0.93 95% CI (0.67–0.997)). (TIF 1258 kb)

## Abbreviations

AUC: Area under the curve
CS: Cardiogenic shock
F_50_: Decreasing the veno-arterial extracorporeal membrane oxygenation flow to 50% of the baseline flow
ICU: Intensive care unit
IDF: Incident dark field
LVEF: Left ventricular ejection fraction
NSW: Not successfully weaned
PPV: Proportion of perfused vessels
PVD: Perfused vessel density
PVDss: Single-spot measurements of perfused vessel density
PVDss_F50_: Single-spot measurements of perfused vessel density during 50% flow reduction
ROC: Receiver operating characteristic
SDF: Sidestream dark field
ss: Single spot
SW: Successfully weaned
TAPSE: Tricuspid annular planer systolic excursion
TDSa: Tissue Doppler lateral mitral annulus peak systolic velocity
TVD: Total vessel density
TVDss: Single-spot measurements of total vessel density
TVDss_F50_: Single-spot measurements of total vessel density during 50% flow reduction
VA-ECMO: Veno-arterial extracorporeal membrane oxygenation
VTI: Aortic velocity-time-integral
VTI_F50_: Aortic velocity–time integral at 50% flow reduction
WA: Weaning attempt
