# Supplementary material for: Functional evaluation of sublingual microcirculation indicates successful weaning from VA-ECMO in cardiogenic shock
Source: Crit Care. 2017 Oct 26;21:265. doi: 10.1186/s13054-017-1855-2 (PMC5658964; doi:10.1186/s13054-017-1855-2)
Supplement: Supplementary file 1 — Shows technical overview of the SDF and IDF devices (adapted with permission from van Elteren et al. [19]). (DOCX 12 kb) [file 13054_2017_1855_MOESM1_ESM.docx]

## Additional file 1

## Table S1: Technical overview of the SDF and IDF devices (adapted with permission from van Elteren et al.^19^)

|  | Sidestream Dark Field | Incident Dark Field |
| --- | --- | --- |
| Dimensions |  |  |
| Length (mm) | 206 | 190 |
| Diameter (mm) | 64 | 28 |
| Weight (g) | 347 | 110 |
| Sensor |  |  |
| Pixel size (µm) | 6.25 x 6.25 | 1.4 x 1.4 |
| Number of megapixel | 0.43 | 14.6 |
| Pulse time (ms) | 16 | 2 |
| Optics |  |  |
| Resolution (lines per mm) | 220 | 320 |
| Magnification | 5 | 4 |
| Field of view (mm2) | 0.84 | 1.79 |
| Focus range (µm) | 0-400 | 0-400 |
