# Supplementary figures and images for: Functional evaluation of sublingual microcirculation indicates successful weaning from VA-ECMO in cardiogenic shock
Source: Crit Care. 2017 Oct 26;21:265. doi: 10.1186/s13054-017-1855-2 (PMC5658964; doi:10.1186/s13054-017-1855-2)

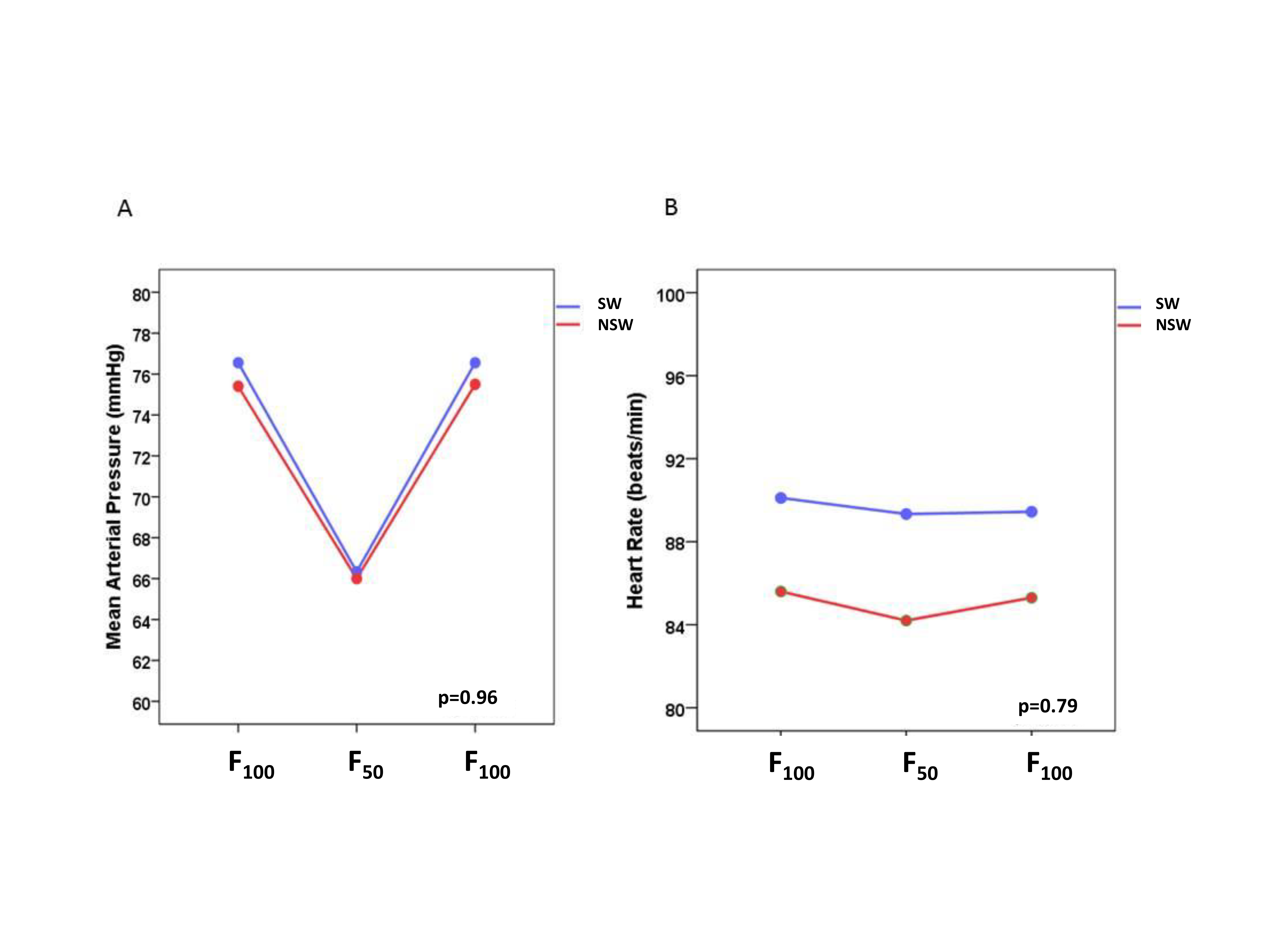

Supplement: Supplementary file 2 — a Mean arterial pressure (MAP) and b heart rate (HR) in patients successfully weaned (SW) and not successfully weaned (NSW). (TIF 2047 kb) [file 13054_2017_1855_MOESM2_ESM.tif]

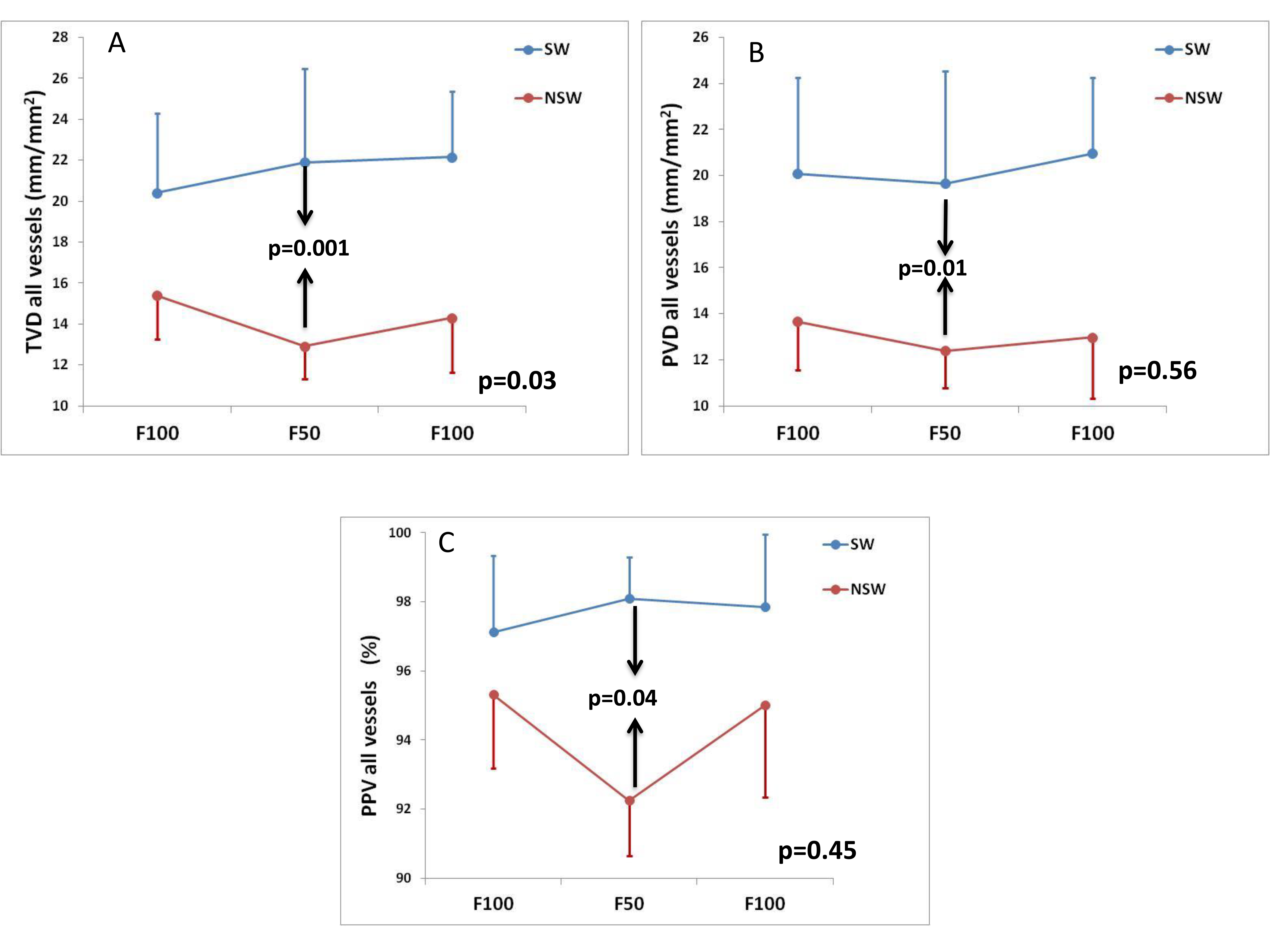

Supplement: Supplementary file 3 — a Total vessel density (TVD), b perfused vessel density (PVD) and c portion of the perfused vessels (PPV) in all vessels (length between 25 and 100 μm) at flow time points of 100% ECMO flow (F100) and 50% ECMO flow (F50) are compared between patients successfully and not successfully weaned (SW and NSW, respectively). (TIF 3043 kb) [file 13054_2017_1855_MOESM3_ESM.tif]

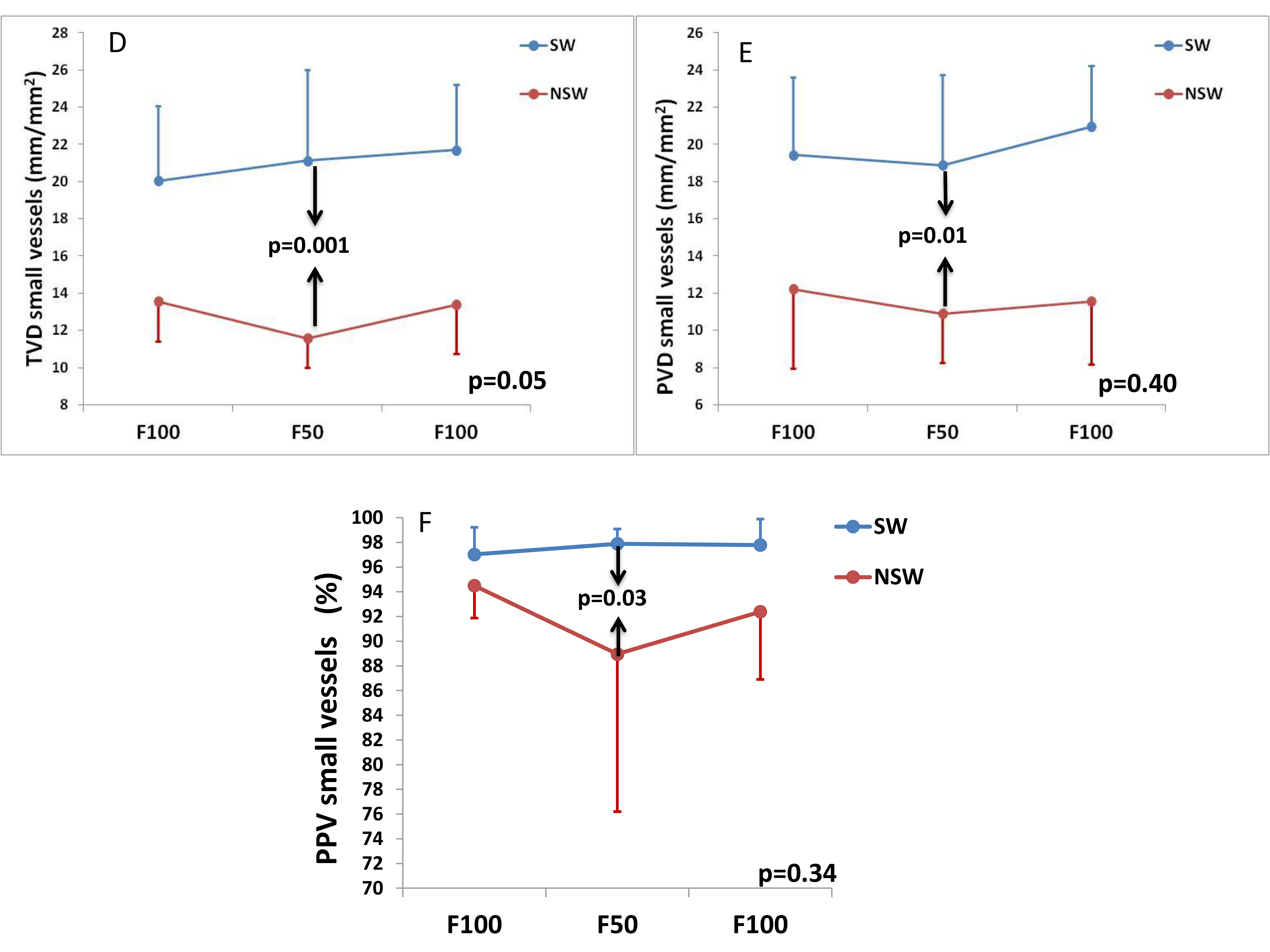

Supplement: Supplementary file 4 — a TVD, b PVD and c PPV in small vessels (capillaries < 25 μm) were compared between each patient during weaning attempts at flow time points of 100% ECMO flow (F100) and 50% ECMO flow (F50) are compared between patients successfully and not successfully weaned (SW and NSW, respectively). (TIF 2730 kb) [file 13054_2017_1855_MOESM4_ESM.tif]

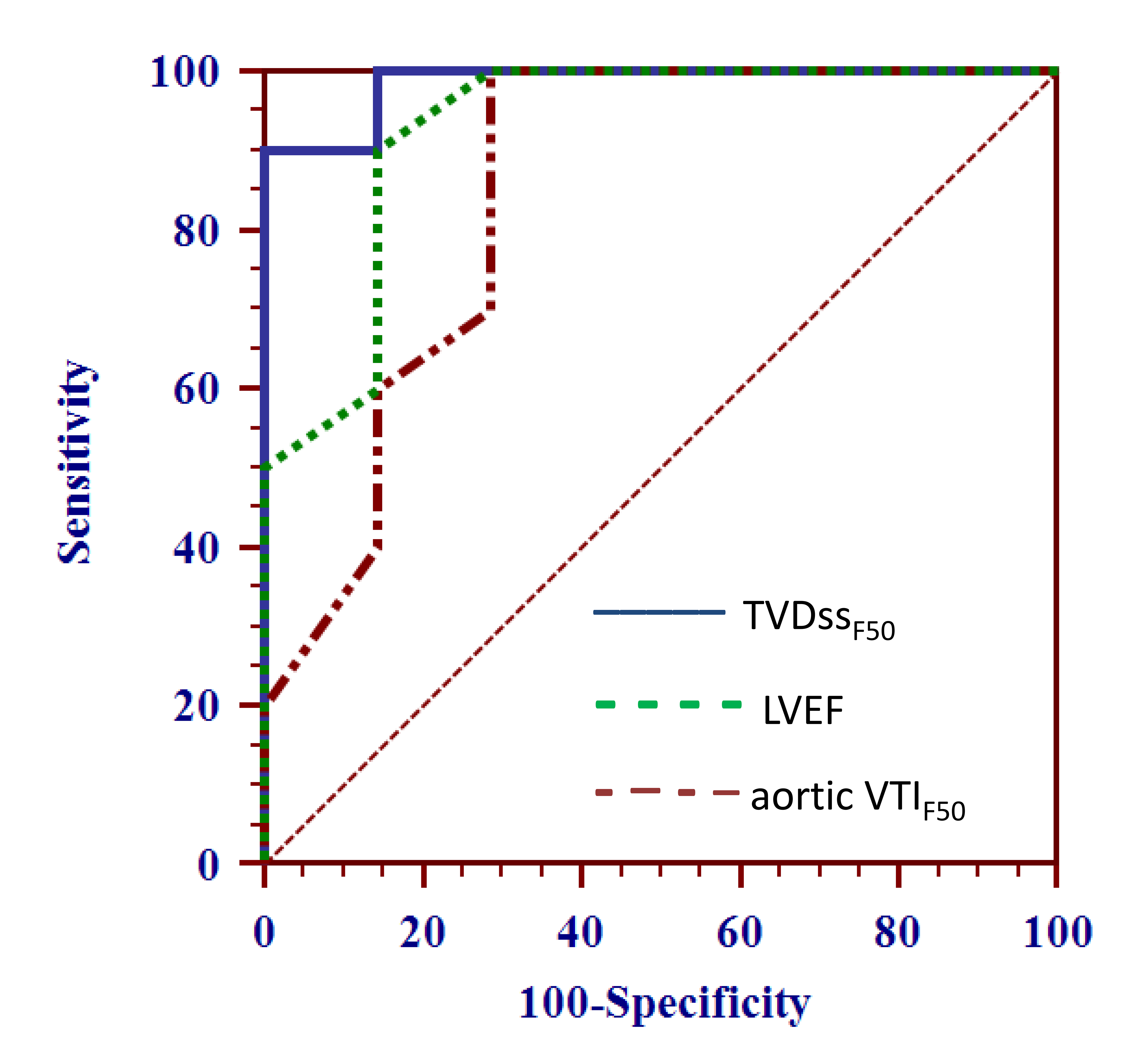

Supplement: Supplementary file 9 — Receiver operating characteristic (ROC) curves for significantly different values from microcirculation and echocardiography as the best parameter from microcirculation and echocardiography according to the area under the ROC curve (AUC). A cutoff value of TVDssF50 > 12.2 mm/mm2 (small vessels) has a higher sensitivity, specificity, and AUC (0.99, 95% CI (0.78–1.00) vs 0.85, 95% CI (0.596–0.97)) compared with aortic VTI F50 > 11 cm and compared with LVEF >15% (0.99, 95% CI (0.78–1.00) vs 0.93 95% CI (0.67–0.997)). (TIF 1258 kb) [file 13054_2017_1855_MOESM9_ESM.tif]
