# Supplementary material for: Functional evaluation of sublingual microcirculation indicates successful weaning from VA-ECMO in cardiogenic shock
Source: Crit Care. 2017 Oct 26;21:265. doi: 10.1186/s13054-017-1855-2 (PMC5658964; doi:10.1186/s13054-017-1855-2)
Supplement: Supplementary file 7 — Shows microcirculatory changes during weaning attempts. Table S2B. Shows microcirculatory differences at baseline VA-ECMO flow. Table S2C. shows microcirculatory differences between baseline and reduced VA-ECMO flow. Table S3. Shows correlation between microcirculatory and echocardiographic parameters for the prediction of successful weaning from VA-ECMO. Table S4A. Shows comparison of the ROC curves of microcirculatory and echocardiographic parameters for the prediction of successful weaning from VA-ECMO. Table S4B. Shows comparison of the ROC curves of microcirculatory and echocardiographic parameters for the prediction of successful weaning from VA-ECMO. (DOCX 27 kb) [file 13054_2017_1855_MOESM7_ESM.docx]

| **Parameters** | **Successfully weaned (n=10)** | | | | **Not successfully weaned (n=3)** | | | |
| --- | --- | --- | --- | --- | --- | --- | --- | --- |
|  | F100 | F50 | F100 | *p-value | F100 | F50 | F100 | *p-value |
| **TVD** |  |  |  |  |  |  |  |  |
| *All Vessels* | 20.42 | 21.89 | 22.16 | 0.347 | 15.4 | 12.91 | 14.30 | **0.025** |
| *Small Vessels* | 20.03 | 21.12 | 21.69 | 0.368 | 13.54 | 11.58 | 13.39 | **0.003** |
| **PVD** |  |  |  |  |  |  |  |  |
| *All Vessels* | 20.08 | 19.66 | 20.96 | 0.293 | 13.67 | 12.39 | 12.97 | 0.407 |
| *Small Vessels* | 19.43 | 18.89 | 20.95 | 0.368 | 12.2 | 10.89 | 11.54 | 0.670 |
| **PPV** |  |  |  |  |  |  |  |  |
| *All Vessels* | 97.13 | 98.1 | 97.85 | 0.607 | 95.31 | 92.25 | 95 | 0.648 |
| *Small Vessels* | 97.03 | 97.89 | 97.79 | 0.607 | 95.64 | 91.82 | 94.03 | 0.648 |
| **MFI** |  |  |  |  |  |  |  |  |
| *All Vessels* | 3 | 2.81 | 2.87 | 0.055 | 3 | 2.96 | 3 | 0.646 |
| *Small Vessels* | 3 | 3 | 3 | 0.273 | 3 | 3 | 3 | 0.368 |

## Additional file 7: Table S2A: Microcirculatory changes during weaning attempts.

*Friedman test was used. Bold formatted p-values are statistically significant.

F100 = ECMO flow at 100% (baseline); F50=ECMO flow at 50% from the baseline flow

TVD = Total vessel density in mm/mm^2^

PVD = Perfused vessel density in mm/mm^2^

PPV = Portion of the perfused vessel density in %

MFI = Microvascular flow index in 1 through 3 values

## Table S2B: Microcirculatory differences at baseline VA-ECMO flow.

| **Parameters** | **Successfully weaned (n=10)** | **Not successfully weaned (n=3)** | ***p-value** |
| --- | --- | --- | --- |
|  | F100 | F100 |  |
| **TVD** |  |  |  |
| *All Vessels* | 20.42 | 15.4 | **0.022** |
| *Small Vessels* | 20.03 | 13.54 | **0.013** |
| **PVD** |  |  |  |
| *All Vessels* | 20.08 | 13.67 | **0.022** |
| *Small Vessels* | 19.43 | 12.2 | **0.013** |
| **PPV** |  |  |  |
| *All Vessels* | 97.13 | 95.31 | 0.497 |
| *Small Vessels* | 97.03 | 95.64 | 0.497 |
| **MFI** |  |  |  |
| *All Vessels* | 3 | 3 | 0.613 |
| *Small Vessels* | 3 | 3 | 0.613 |

*Mann Whitney U test was used. Median value was showed. Bold formatted p-values are statistically significant.

F100 = ECMO flow at 100% (baseline); F50=ECMO flow at 50% from the baseline flow

TVD = Total vessel density in mm/mm^2^

PVD = Perfused vessel density in mm/mm^2^

PPV = Portion of the perfused vessel density in %

MFI = Microvascular flow index in 1 through 3 values

## Table S2C: Microcirculatory differences between baseline and reduced VA-ECMO flow.

| **Parameters** | **Successfully weaned (n=10)** | | | **Not successfully weaned (n=3)** | | |
| --- | --- | --- | --- | --- | --- | --- |
|  | F100 | F50 | ***p-value** | F100 | F50 | ***p-value** |
| **TVD** |  |  |  |  |  |  |
| *All Vessels* | 20.42 | 21.89 | 0.953 | 15.4 | 12.91 | **0.007** |
| *Small Vessels* | 20.03 | 21.12 | 0.594 | 13.54 | 11.58 | **0.005** |
| **PVD** |  |  |  |  |  |  |
| *All Vessels* | 20.08 | 19.66 | 0.594 | 13.67 | 12.39 | 0.386 |
| *Small Vessels* | 19.43 | 18.89 | 0.515 | 12.2 | 10.89 | 0.445 |
| **PPV** |  |  |  |  |  |  |
| *All Vessels* | 97.13 | 98.10 | 0.917 | 95.31 | 92.25 | 0.314 |
| *Small Vessels* | 97.03 | 97.89 | 0.917 | 95.64 | 91.82 | 0.260 |
| **MFI** |  |  |  |  |  |  |
| *All Vessels* | 3 | 2.91 | 0.430 | 3 | 2.96 | 0.893 |
| *Small Vessels* | 3 | 3 | 0.180 | 3 | 3 | 0.593 |

*Wilcoxon test was used.

F100 = ECMO flow at 100% (baseline); F50=ECMO flow at 50% from the baseline flow.

Bold formatted p-values are statistically significant.

TVD = Total vessel density in mm/mm^2^

PVD = Perfused vessel density in mm/mm^2^

PPV = Portion of the perfused vessel density in %

MFI = Microvascular flow index in 1 through 3 values

## Table S3: Correlation between microcirculatory and echocardiographic parameters for the prediction of successful weaning from VA-ECMO.

| **Correlation** | **r-value** | **p-value** |
| --- | --- | --- |
| TVD all vessels – LVEF | 0.5327 | **0.02** |
| TVD small vessels-LVEF | 0.6214 | **0.01** |
| PVD all vessels-LVEF | 0.4983 | **0.03** |
| PVD small vessels-LVEF | 0.4816 | **0.04** |

TVD = Total vessel density in mm/mm^2^

PVD = Perfused vessel density in mm/mm^2^

LVEF = Left ventricular ejection fraction in %

Bold formatted p-values are statistically significant.

## Table S4A. Comparison of the ROC curves of microcirculatory and echocardiographic parameters for the prediction of successful weaning from VA-ECMO.

|  | **AUC** | **SE ^a^** | **95% CI ^b^** | **p-value** |
| --- | --- | --- | --- | --- |
| TVDss_F50_ | 0.986 | 0,0202 | 0,780 to 1,000 | (versus LVEF) = 0.45 |
| LVEF | 0.929 | 0,0686 | 0,696 to 0,997 | (versus Aortic VTI_F50_) = 0.23 |
| Aortic VTI_F50_ | 0,850 | 0,110 | 0,596 to 0,974 | (versus TVDss_F50_) = 0.21 |

^a^ DeLong et al., 1988

^b^ Binomial exact

F50=ECMO flow at 50% from the baseline flow

TVDss = Total vessel density measured in the same single-spot in mm/mm^2^

LVEF = Left ventricular ejection fraction in %

VTI = Doppler velocity time integral in cm

## Table S4B. Comparison of the ROC curves of microcirculatory and echocardiographic parameters for the prediction of successful weaning from VA-ECMO.

|  | **AUC** | **SE ^a^** | **95% CI ^b^** | **p-value** |
| --- | --- | --- | --- | --- |
| PVDss_F50_ | 0,914 | 0,0668 | 0,676 to 0,994 | (versus LVEF) = 0.89 |
| LVEF | 0,929 | 0,0686 | 0,696 to 0,997 | (versus Aortic VTI_F50_) = 0.21 |
| Aortic VTI_F50_ | 0,850 | 0,110 | 0,596 to 0,974 | (versus PVDss_F50_) = 0.66 |

^a^ DeLong et al., 1988

^b^ Binomial exact

F50=ECMO flow at 50% from the baseline flow

PVDss = Perfused vessel density measured in the same single-spot in mm/mm^2^

LVEF = Left ventricular ejection fraction in %

VTI = Doppler velocity time integral in cm
