# Supplementary material for: Functional evaluation of sublingual microcirculation indicates successful weaning from VA-ECMO in cardiogenic shock
Source: Crit Care. 2017 Oct 26;21:265. doi: 10.1186/s13054-017-1855-2 (PMC5658964; doi:10.1186/s13054-017-1855-2)
Supplement: Supplementary file 8 — Additionale data. (DOCX 13 kb) [file 13054_2017_1855_MOESM8_ESM.docx]

**Additional file 8**

**Functional evaluation of sublingual microcirculation indicates successful weaning from VA-ECMO in Cardiogenic Shock**

Sakir Akin MD^1,2^, Dinis dos Reis Miranda MD, PhD^1^, Kadir Caliskan MD, PhD^2^,

Osama I. Soliman, MD, PhD ^2^, Goksel Guven ^MD,1,2^, Ard Struijs MD, PhD^1^, Robert J. van Thiel MD^1^,

Lucia S. Jewbali MD^1,2^, Alexandre Lima MD, PhD^1^, Diederik Gommers MD, PhD^1^, Felix Zijlstra MD, PhD^2^ and Can Ince PhD^1^

**Legends to additional data**

**Additional file 5: Additional Clip 1** A patient with successful weaning attempt. Microcirculatory image clips recorded during a weaning attempt.

**Additional file 6: Additional Clip 2** A patient with non-successful weaning attempt. Microcirculatory image clips recorded during a weaning attempt.

**Additional file 2: Figure 1: A:** Mean arterial pressure (MAP) and **B:** heart rate (HR) in patients successfully weaned (SW) and not successfully weaned (NSW) .

F_100_ is the baseline measurement at an ECMO flow of 100%. F_50_ is the time point at 50% of the baseline ECMO flow.

**Additional file 3: Figure 2 A, B and C:** The Total Vessel Density **(**TVD), **B:** Perfused Vessel density (PVD) and **C:** Portion of the Perfused Vessels (PPV) in all vessels (length between 25-100 µm) at flow time points of 100% ECMO flow (F100) and 50% ECMO flow (F50) are compared between patients successfully and not successfully weaned (SW and NSW, respectively).

**Additional file 4: Figure 2 D, E and F**. D: TDV, E: PVD and F: PPV of small vessels (capillaries < 25 µm) were compared between each patient during weaning attempts at flow time points of 100% ECMO flow (F100) and 50% ECMO flow (F50) are compared between patients successfully and not successfully weaned (SW and NSW, respectively).

**Additional file 9: Figure 3:** Receiver operating characteristic (ROC) curves for significantly different values from microcirculation and echocardiography as the best parameter from microcirculation and echocardiography according to the area under the ROC curve (AUC). A cut-off value of TVDss_F50_ >12.2 mm/mm^2^ (small vessels) has a higher sensitivity, specificity, and AUC (0.99, 95% CI [0.78-1.00] vs 0.85, 95% CI [0.596-0.97]) compared with aortic VTI _F50_ >11 cm and compared with LVEF > 15% (0.99, 95% CI [0.78-1.00] vs 0.93 95% CI [0.67-0.997])
